# Clemastine Ameliorates Myelin Deficits *via* Preventing Senescence of Oligodendrocytes Precursor Cells in Alzheimer’s Disease Model Mouse

**DOI:** 10.3389/fcell.2021.733945

**Published:** 2021-10-21

**Authors:** Yuan-Yuan Xie, Ting-Ting Pan, De-en Xu, Xin Huang, Yong Tang, Wenhui Huang, Rui Chen, Li Lu, Hao Chi, Quan-Hong Ma

**Affiliations:** ^1^Department of Neurology and Clinical Research Center of Neurological Disease, The Second Affiliated Hospital of Soochow University, Suzhou, China; ^2^Jiangsu Key Laboratory of Neuropsychiatric Diseases, Institute of Neuroscience, Soochow University, Suzhou, China; ^3^Department of Anatomy, Shanxi Medical University, Taiyuan, China; ^4^Wuxi No. 2 People’s Hospital, Wuxi, China; ^5^International Collaborative Centre on Big Science Plan for Purinergic Signaling, Chengdu University of Traditional Chinese Medicine, Chengdu, China; ^6^Acupuncture and Chronobiology Key Laboratory of Sichuan Province, Chengdu, China; ^7^Molecular Physiology, Center for Integrative Physiology and Molecular Medicine (CIPMM), University of Saarland, Homburg, Germany

**Keywords:** clemastine, Alzheimer’s disease, myelin, oligodendrocyte precursor cells, oligodendrocytes, cellular senescence

## Abstract

Disrupted myelin and impaired myelin repair have been observed in the brains of patients and various mouse models of Alzheimer’s disease (AD). Clemastine, an H1-antihistamine, shows the capability to induce oligodendrocyte precursor cell (OPC) differentiation and myelin formation under different neuropathological conditions featuring demyelination *via* the antagonism of M1 muscarinic receptor. In this study, we investigated if aged APPSwe/PS1dE9 mice, a model of AD, can benefit from chronic clemastine treatment. We found the treatment reduced brain amyloid-beta deposition and rescued the short-term memory deficit of the mice. The densities of OPCs, oligodendrocytes, and myelin were enhanced upon the treatment, whereas the levels of degraded MBP were reduced, a marker for degenerated myelin. In addition, we also suggest the role of clemastine in preventing OPCs from entering the state of cellular senescence, which was shown recently as an essential causal factor in AD pathogenesis. Thus, clemastine exhibits therapeutic potential in AD *via* preventing senescence of OPCs.

## Introduction

Clemastine fumarate is a first-generation H1-antihistamine that is mainly used for relieving symptoms of allergic reactions primarily by competing with histamine to bind H1 receptors (Blazsó and Gábor, 1997). In addition to its anti-inflammatory effects, clemastine also shows antimuscarinic type-1 receptors effects. In recent years, it was also found clemastine, among other antimuscarinic compounds, can significantly induce oligodendrocyte precursor cell (OPC) differentiation and myelination (Mei et al., 2014), accelerating the remyelination process in animal models featured by demyelination (Mei et al., 2014, 2016; Li et al., 2015; Cree et al., 2018). Furthermore, a phase 2 clinical trial on using clemastine for treating multiple sclerosis (MS) showed that remyelination could be achieved during the chronic neurodegeneration process (Green et al., 2017). These studies collectively strongly suggest clemastine is a potent stimulator of myelin formation with a good safety profile. However, the precise mechanisms of clemastine promoting remyelination are still unknown.

Substantial myelin impairment was also observed in Alzheimer’s disease (AD) (Dean et al., 2017; Papuć and Rejdak, 2018). A significant reduction of ceramide synthase 2 activity, an enzyme necessary for myelin synthesis, was observed in the early stage of AD, before the appearance of neurofibrillary tangles (Couttas et al., 2016). Even for the patients suffering from amnestic mild cognitive impairment, which is considered as prodromal AD, significant myelin reduction was detected, indicating demyelination is initiated at an early stage during the pathogenesis process (Carmeli et al., 2013). Moreover, focal demyelination was also found to be associated with amyloid-beta (Aβ) plaques (Mitew et al., 2010), which further highlights the association between demyelination and AD. In the etiology of the demyelination, recent studies suggest that the senescence of OPCs is a major contributor to the demyelination for both AD and MS (Nicaise et al., 2019; Zhang et al., 2019). Albeit our understanding of the role of myelin changes in AD is still poor, as to whether it is a critical event in AD is still unclear (Papuć and Rejdak, 2018), clemastine could provide us a new way to study the impact of myelin changes in AD.

In our previous study, we found that chronic treatment of clemastine reduces brain Aβ deposition and rescues the cognitive deficits of 7-month-old APPSwe/PS1dE9 (APP/PS1) mice (Li et al., 2021). Consistent with that, another recent study also shows that chronic treatment of clemastine can improve the memory of young APP/PS1 mice and induces significant myelin formation concomitantly (Chen et al., 2021). In this study, we investigated if chronic treatment of clemastine has therapeutic effects for aged APP/PS1 mice. After 2 months of clemastine treatment for 15-month-old APP/PS1 mice, we found that the Aβ depositions in APP/PS1 mice brain is reduced, and the short-term memory is also rescued. Furthermore, clemastine shows strong protective effects for OPCs, as it spurs the proliferation and differentiation of OPCs and the myelin formation. In addition, our data also indicate that the protective effects could be attributed to its ability to prevent OPCs from entering the state of cellular senescence. Taken together, these results illustrated the therapeutic effects of clemastine for aged APP/PS1 mice, indicating clemastine potentially could provide beneficial effects for AD patients.

## Materials and Methods

### Animals

APP/PS1 transgenic mice (stock number 004462) were purchased from the Jackson Laboratory and maintained by breeding with C57BL/6 mice. Male APP/PS1 transgenic mice and the age-matched wild-type (WT) mice were used in all experiments. Animal care and surgical procedures were approved by the Institutional Animal Care and Use Committee of Soochow University in accordance with international laws.

### Drug Treatments

Fifteen-month-old APP/PS1 transgenic mice received a diet of standard laboratory chow supplemented with clemastine (10 mg/kg/day) (sodium salt, Tocris Bioscience) for 2 months (Li et al., 2021). The transgenic mice and the WT mice received the same chow without clemastine.

### Immunoblotting

Lysates from the cortex and hippocampus were extracted in lysis buffer [50-mM Tris (pH 7.4), 150-mM sodium chloride, 1% TritonX-100, 1% sodium deoxycholate, 0.1% sodium dodecyl sulfate] containing protease and phosphatase inhibitor cocktail (Roche, Switzerland). Lysates were subjected to sodium dodecyl sulfate-polyacrylamide gel electrophoresis, then transferred to a polyvinylidene fluoride membrane (Millipore, Darmstadt, Germany). The membrane was blocked in 5% bovine serum albumin dissolved in Tris-buffered saline containing Tween 20 (TBST) (20-mM Tris-HCL pH 7.6, 150-mM sodium chloride, and 0.1% Tween 20) for 1 h at room temperature (RT) and then was incubated with the appropriate primary antibodies in 5% bovine serum albumin diluted in TBST for overnight at 4°C. The membranes were washed with TBST and incubated with horseradish peroxidase-conjugated secondary antibodies (Sigma-Aldrich, United States) for 1 h at RT. The immunoreacted proteins were visualized using ECL Western Blotting Detection Reagents (Pierce, United States). Semiquantitative analysis of band density was performed in ImageJ^1^ for the protein levels measurement. A list of the primary antibodies used in this study can be found in Supplementary Table 1.

### Immunostaining

The 17-month-old mice were anesthetized with 1% pentobarbital and perfused with phosphate-buffered saline (PBS), followed by fixation with 4% paraformaldehyde (PFA) overnight. After that, the brain tissues were dehydrated and embedded in an optimal cutting temperature compound. Coronal cryosections of hippocampal tissue and the cortex were collected. In contrast, for the cultured cells, the cells were rinsed with cold PBS before being fixed with 4% PFA for 15 min on ice. In immunostaining, the brain sections or the fixed cells were washed with PBS containing 0.5% Triton and blocked with immunostaining blocking buffer (P0102, Beyotime) for 1 h. Subsequently, the sections are incubated with primary antibodies overnight at 4°C. After rinsing in PBS, the sections are incubated with a secondary antibody for 1 h at RT. The sections are subsequently mounted with the mounting medium containing 4,6-diamidino-2-phenylindole (Vector Laboratories). Images were captured under a Leica confocal microscope LSM700.

### Novel Object Recognition Test

A novel object recognition test was performed in a 40 × 40 × 40-cm open arena. The behavior of the mouse was recorded by a camera above and analyzed in the software ANY-maze. Two identical objects were placed on opposite sides in the familiarization phase. In a 10-min time frame, the time of the mice exploring the two objects was recorded. An object preference index is then calculated, which equals the time exploring one of the identical objects/time exploring the identical object pairs × 100%. If the mice show no difference on the object preference index, then one of the objects is replaced with a novel object, and the test phase is carried out. Recognition index is calculated accordingly, which equals the time exploring novel object/(time exploring novel object + time exploring familiar object) × 100%.

### Morris Water Maze Test

In the learning phase, the mice took four trials per day for six consecutive days. A different starting position was used on each trial. The duration of one trial was 90 s for the mice to find the platform. Escape latencies (time spent swimming from the start point to the target) and path length (the distance from the start point to the platform), as well as swimming speed, were recorded. The escape latencies in the following training day were analyzed (escape latency in the following day/escape latency in the first day) and labeled as a learning trend. For probe trials, the platform was removed after the last trial of the learning phase. Mice were tested 24 h later to assess memory consolidation. The time spent in the target quadrant within 60 s was recorded. The latency to the first target site was measured, and the numbers of platform-site crossovers were recorded.

### Myelin Isolation

The central nervous system myelin isolation method is based on the previous publication (Erwig et al., 2019). Adult Sprague–Dawley rats were perfused with PBS, and the brain was homogenized in 0.32-M sucrose. The brain lysates were laid on 0.85-M sucrose and centrifuged at 75,000 × *g* for 30 min. The crude myelin presented at the interface was harvested and resuspended in dH_2_O, then centrifuged at 75,000 × *g* for 30 min. The myelin pellet after the centrifuge is collected and washed once with ddH_2_O at 12,000 × *g* for 15 min. The myelin pellet after the centrifuge was resuspended in 0.32-M sucrose, laid on 0.85-M sucrose, and centrifuged at 75,000 × *g* for 30 min. The myelin pellet was collected after the centrifuge and resuspended in ddH_2_O. Centrifuged at 75,000 × *g* for 15 min, the purified myelin pellet is collected and resuspended in TBS and stored at –80°C. All the isolation procedures were carried out on ice or at 4°C during the centrifuges.

The oligodendrocyte cell line (OLN) cells were treated with the isolated myelin (10 μg/ml) for 30 min to induce cellular senescence in immunostainings or treated with the isolated myelin (30 μg/ml) for 4 h in Western blot analysis. After that, the cells were treated with 3-μM clemastine (dissolved in dimethyl sulfoxide) for 12 h. The cells treated with dimethyl sulfoxide is served as control. Then, cells were fixed in 4% PFA for 15 min on ice.

### Senescence-Associated β-Galactosidase Staining

Senescence-associated β-galactosidase (SA-β-gal) staining was performed for both the fixed cells and the brain cryosections according to the manufacturer’s instructions (CellEvent^TM^ Senescence Green Detection Kit, Invitrogen), whereas the sections were subsequently subjected for immunostaining of platelet-derived growth factor-α (PDGFRα). Images were captured under a Leica confocal microscope LSM700.

### Statistics Analysis

All the imaging data were processed in Image J and subsequently analyzed in SPSS 25.0 and GraphPad Prism 8.0. All the data are shown as mean ± standard error of the mean. One-way analysis of variance was used to compare the difference between multiple groups. Unpaired Student’s *t*-test was used to analyze the difference between the two groups. *P*-value indicates significance, and *P* < 0.05 indicates statistical significance.

To calculate the density of OPCs and oligodendrocytes (OLs) (PDGFRα or CC1 immuno-positive cells per square millimeter), the number of the cells was quantified. Then, it is divided by the surface area of the brain section (square millimeter) to calculate the cell density. The percentage of the Aβ covering area is calculated in a similar way. The Aβ covering area is measured, and then, it is divided by the surface area of the brain section (square millimeter). To quantify the percentage of PDGFRα^+^ SA-β-gal^+^ and PDGFRα^+^ p21^+^ cells, the number of double-positive cells and the total PDGFRα^+^ cells were quantified. Then, the number of double-positive cells is divided by the total PDGFRα^+^ cells to calculate the percentage.

## Results

### Chronic Treatment of Clemastine Rescues the Short-Term Memory Deficit of Aged APP/PS1 Mice

**FIGURE 1:**
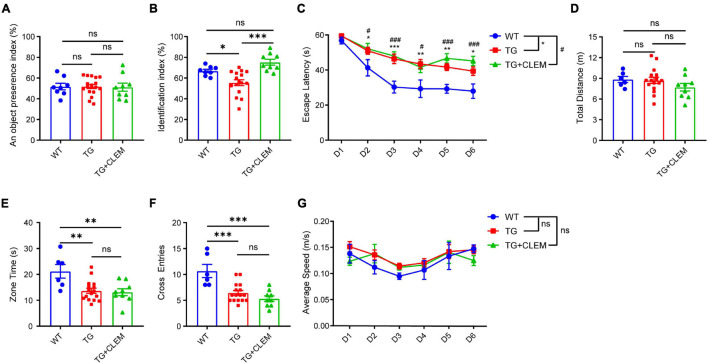
Chronic treatment of clemastine rescues short-term memory deficit of aged APP/PS1 mice. Analyses of mice behavior in novel object recognition test **(A,B)** and Morris water maze **(C–G)**. Index of each analysis includes: **(A)** object preference index, **(B)** identification index, **(C)** escape latency, **(D)** total distance (m), **(E)** zone time and **(F)** cross entries, **(G)** average speed (m/s), should refers to Methods for detail. WT: Wild-type mice. TG: Transgenic mice (APP/PS1). TG + CLEM: Transgenic mice (APP/PS1) treated with clemastine. All tested mice were 17 months old. Values shown represent mean ± SE. ^*#^*p* < 0.05, ^∗∗^*p* < 0.01, ^***###^*p* < 0.001, *n* = 8–17 mice per group, one-way analysis of variance with Student’s *t*-test.

To investigate if the treatment of clemastine could have a therapeutic effect on aged APP/PS1 mice, clemastine was orally administrated to 15-month-old APP/PS1 mice for 2 months. We first examined the impact of clemastine treatment on cognitive functions to see if it can rescue the cognitive deficits of the 17-month-old APP/PS1 mice. To evaluate the memory of the mice, we performed both a novel object recognition test and Morris water maze. In a novel object recognition test, we confirmed that the age-matched WT mice and APP/PS1 mice treated with or without clemastine show no difference on preference index during the familiarization phase (Figure 1A). In contrast, in the test phase, we found that APP/PS1 mice show reduced identification index in comparison with the WT mice (Figure 1B), and the treatment of clemastine rescues the index value (Figure 1B). This result indicates that the short-term memory deficits of the aged APP/PS1 mice can be rescued by clemastine treatment. In the Morris water maze, the APP/PS1 mice display longer escape latency than the WT mice during the learning phase, indicating that the learning ability is impaired for the APP/PS1 mice (Figure 1C). In the probe trial, despite that the swimming distance shows no differences (Figure 1D), the APP/PS1 mice spent a shorter time in the target quadrant and swam crossover the target site less often than the WT mice, which indicates the memory retention is impaired for the APP/PS1 mice (Figures 1E,F). However, the treatment of clemastine did not alter the performance of the APP/PS1 mice in the trial (Figures 1C–F). As all the groups showed no differences in swimming speed, the swimming ability is not a factor affecting the performances of the mice (Figure 1G). Together, these data suggest that clemastine treatment can rescue the short-term memory impairment but not the memory retention deficits or learning ability impairment of the aged APP/PS1 mice.

### Chronic Treatment of Clemastine Ameliorates the Accumulation of Amyloid-β in the Aged APP/PS1 Mice Brain

**FIGURE 2:**
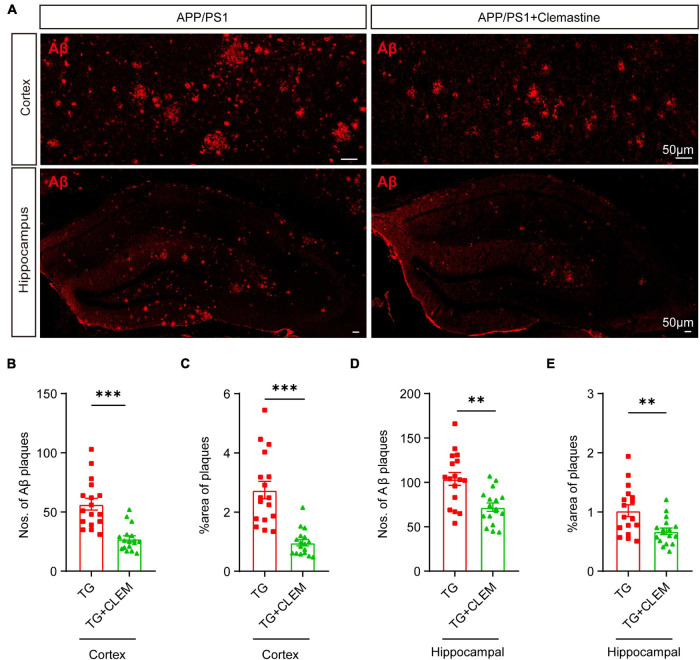
Chronic treatment of clemastine ameliorates accumulation of Aβ in aged APP/PS1 mice brain. **(A)** Confocal images of immunolabeling of Aβ (red) in hippocampus/cortex of APP/PS1 and APP/PS1 mice treated with clemastine. **(B–E)** Quantification of numbers of Aβ plaque and percentage of Aβ plaque covering areas of hippocampus **(B,C)**/cortex **(D,E)** as shown in **(A)**. All brain sections were collected from 17-month-old mice. Values shown represent mean ± SE. ^∗∗^*p* < 0.01, ^∗∗∗^*p* < 0.001, *n* = 4 mice per group, one-way analysis of variance with Student’s *t*-test.

Next, we examined the accumulation of Aβ in the brain to evaluate the brain neuropathological burden. A previous study showed that large Aβ plaques were formed in APP/PS1 mice at 10 months of age, which became even larger and covered more area at 17 months of age (Rupp et al., 2011). Consistent with the previous finding, by immunostaining against Aβ of 17-month-old APP/PS1 mice brain, we also observed that large Aβ plaques were formed in both the cortex and hippocampus (Figure 2A). In contrast, treatment of clemastine significantly reduced the numbers and the covering areas of Aβ plaques (Figure 2). For the cortex, on average, there was over a 50% reduction of the Aβ plaque numbers and the covering area of it (Figures 2B,C). In contrast, for the hippocampus, there was approximately 30% reduction of the Aβ plaque numbers and the covering area of it (Figures 2D,E). These data show that the brain neuropathological burden was greatly alleviated upon the chronic treatment of clemastine for only 2 months, even at the late stage of the disease.

### Chronic Treatment of Clemastine Prevents the Loss of Oligodendrocyte Precursor Cells and Oligodendrocytes in Aged APP/PS1 Mice

**FIGURE 3:**
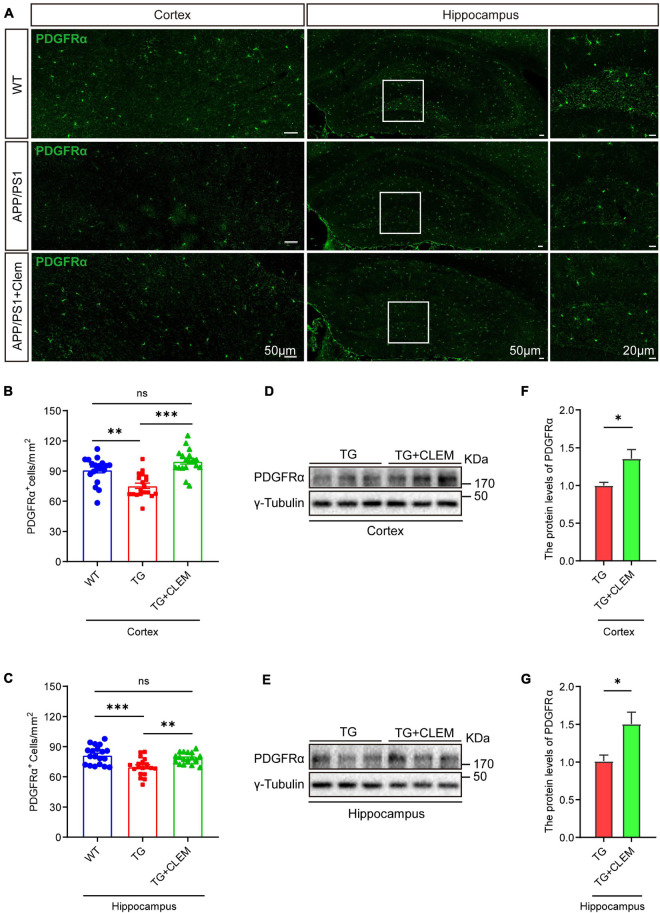
Chronic treatment of clemastine increases number of OPCs in aged APP/PS1 mice brain. **(A)** Confocal images of immunolabeling of PDGFRα (green) in hippocampus/cortex of WT; APP/PS1 and APP/PS1 mice treated with clemastine. White box indicates magnified area of hippocampus. **(B,C)** Quantification of number of PDGFRα^+^ cells in hippocampus **(B)** and cortex **(C),** respectively. **(D,E)** Western analysis of PDGFRα levels in hippocampus **(D)** or cortex **(E)** of APP/PS1 or APP/PS1 mice treated with clemastine; γ-tubulin was served as loading control. **(F,G)** Quantification of replicated results shown in **(D,E)**, respectively. Ratio values were calculated as “PDGFRα/γ-Tubulin” and normalized to ratio of APP/PS1 mice. Values shown represent mean ± SE. ^∗^*p* < 0.05, ^∗∗^*p* < 0.01, ^∗∗∗^*p* < 0.001, *n* = 4 mice per group for **(B,C)** and *n* = 3 mice per group for **(F,G)**, one-way analysis of variance with Student’s *t*-test.

**FIGURE 4:**
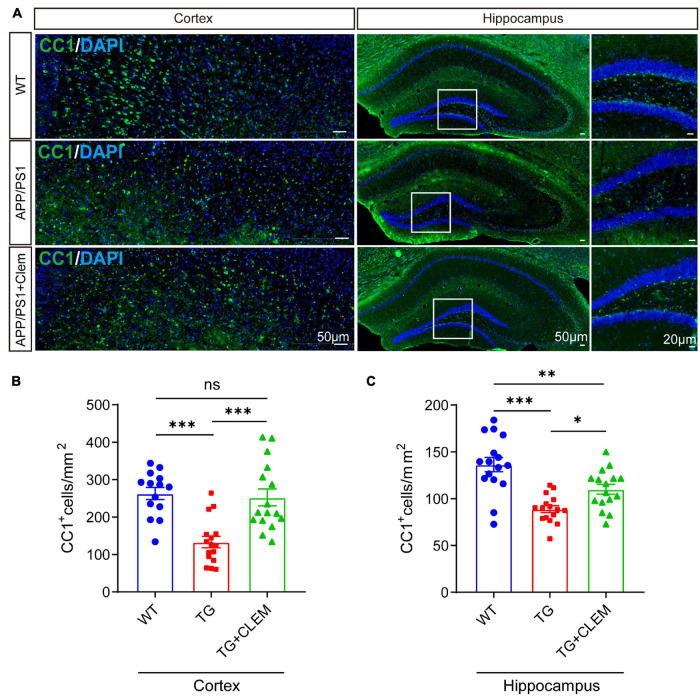
Chronic treatment of clemastine increases number of OLs in aged APP/PS1 mice brain. **(A)** Confocal images of immunolabeling of CC1 (green) and 4,6-diamidino-2-phenylindole (blue) in hippocampus/cortex of WT; APP/PS1 and APP/PS1 mice treated with clemastine. White box indicates magnified area of hippocampus. **(B,C)** Quantification of number of CC1^+^ cells in hippocampus **(B)** and cortex **(C)**, respectively. Values shown represent mean ± SE. ^∗^*p* < 0.05, ^∗∗^*p* < 0.01, ^∗∗∗^*p* < 0.001, *n* = 4 mice per group, one-way analysis of variance with Student’s *t*-test.

To understand the mechanisms of the neurological amelioration effects of clemastine on APP/PS1 mice, we first investigated the impact of clemastine on modulating the proliferation of OPCs. By immunolabeling PDGFRα, a marker of OPCs (Li et al., 2017), we found that there was a significant decline of the PDGFRα immuno-positive(^+^) cells of the APP/PS1 mice in comparison with the WT mice in both the hippocampus and the cortex (Figures 3A–C), whereas the treatment of clemastine restored the number of PDGFRα^+^ cells to the level of WT mice (Figures 3A–C). Consistent with that, through Western blot analysis, we also observed an increase of PDGFRα level for the APP/PS1 mice treated with clemastine (Figures 3D–G). Therefore, clemastine prevents the loss of OPCs in the brains of aged APP/PS1 mice. By immunolabeling differentiated OLs using the CC1 clone against adenomatous polyposis coli (Bin et al., 2016), we observed an over 50 and 30% decline of CC1^+^ cells in the cortex and hippocampus, respectively, of the APP/PS1 mice in comparison with the WT mice (Figure 4), whereas the treatment of clemastine increased the numbers of CC1^+^ OLs in those two regions (Figure 4). Thereby, the clemastine treatment prevents the loss of OPCs and OLs in the brains of the aged APP/PS1 mice.

### Chronic Treatment of Clemastine Facilitates the Formation of Myelin, Preventing Its Degeneration in Aged APP/PS1 Mice

**FIGURE 5:**
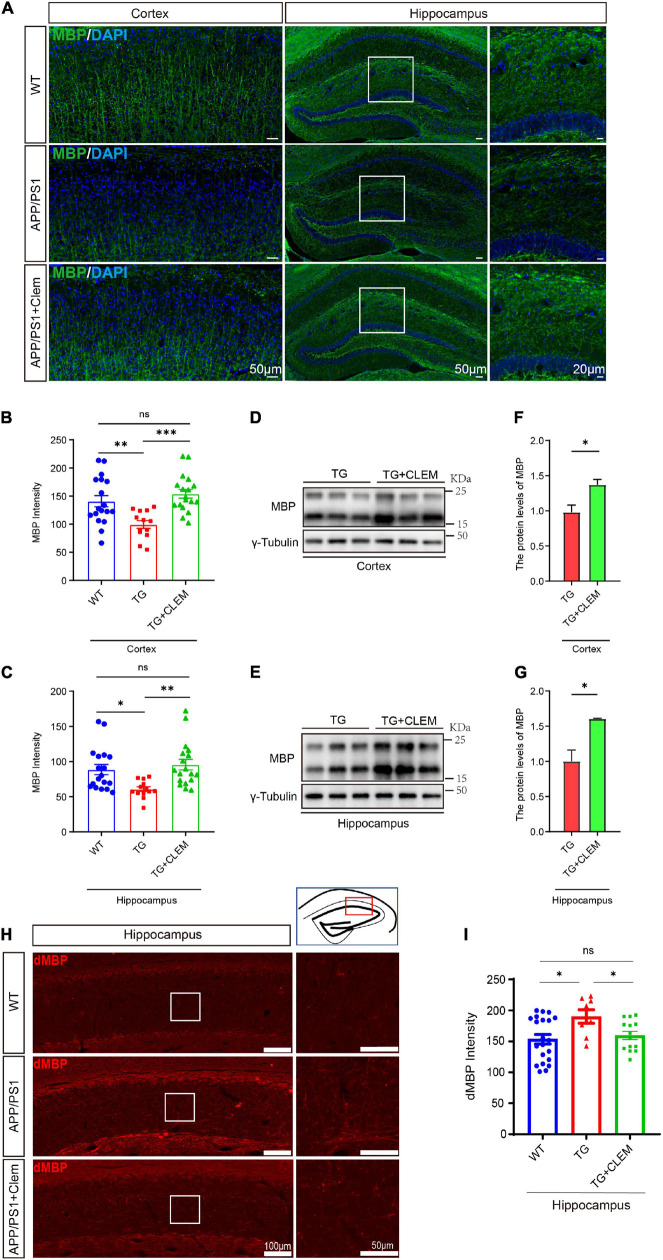
Chronic treatment of clemastine enhances myelin formation in aged APP/PS1 mice brain. **(A)** Confocal images of immunolabeling MBP (green) in hippocampus/cortex of WT; APP/PS1 and APP/PS1 mice treated with clemastine. **(B,C)** Quantification of MBP signal intensity in cortex **(B)** or hippocampus **(C)** from immunostaining. **(D,E)** Western analysis of MBP levels in cortex **(D)** or hippocampus **(E)** of APP/PS1 or APP/PS1 mice treated with clemastine; γ-tubulin was served as loading control. **(F,G)** Quantification of replicated results shown in **(D,E)**, respectively. **(H)** Confocal images of immunolabeling dMBP in hippocampus of WT; APP/PS1 and APP/PS1 mice treated with clemastine. White box indicates magnified area of hippocampus. **(I)** Quantification of dMBP signal intensity shown in **(H)**. Values shown represent mean ± SE. ^∗^*p* < 0.05, ^∗∗^*p* < 0.01, ^∗∗∗^*p* < 0.001, *n* = 4 mice per group for **(B,C,H)** and *n* = 3 mice per group for **(F,G)**, one-way analysis of variance with Student’s *t-*test.

We wonder if an increase in myelin formation accompanies the increased numbers of OPCs and OLs. By immunolabeling myelin basic protein (MBP), the main protein component of myelin (Liu et al., 2021), we found that compared with the WT mice, the MBP immunofluorescence intensity was decreased for the APP/PS1 mice (Figures 5A–C), which was reverted by the treatment of clemastine (Figures 5A–C). Consistently, in Western blot analysis, we also detected the MBP protein level increased upon the treatment of clemastine (Figures 5D–G). Thus, the clemastine treatment induced myelin formation in the aged APP/PS1 mice. On the other hand, we evaluated myelin degeneration by immunolabeling degraded myelin basic protein complex (dMBP), which specifically reflects the degenerating myelin (Matsuo et al., 1997). A significant increase of dMBP signal was observed for the APP/PS1 mice in comparison with WT mice in the hippocampus, whereas the clemastine treatment reduced the signal of dMBP (Figures 5H,I), indicating the treatment that protected OL MBP from degenerating. Therefore, clemastine treatment enhanced the myelin formation and prevented its degradation in aged APP/PS1 mice.

### Clemastine Treatment Prevents the Senescence of Oligodendrocyte Precursor Cells

**FIGURE 6:**
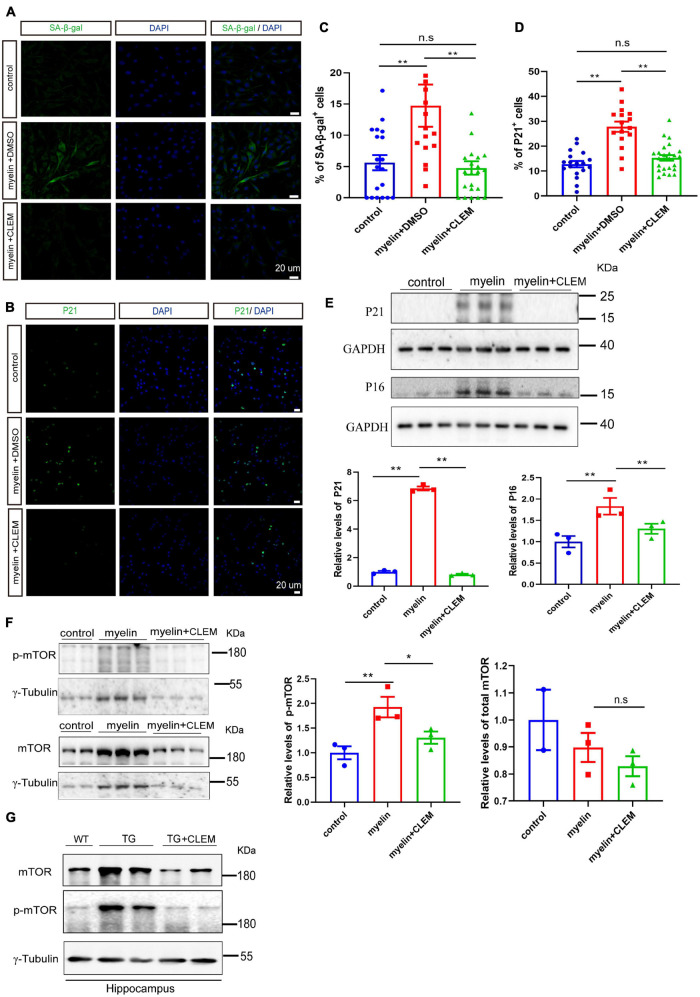
Clemastine treatment prevents senescence of cultured glial cells. **(A,B)** Confocal images of immunolabeling OLN cells with SA-β-gal **(A)** or p21 **(B)** (green) and 4,6-diamidino-2-phenylindole (blue). Cells were either non-treated (control) or treated with myelin and dimethyl sulfoxide (myelin) or treated with myelin and clemastine (myelin + CLEM). **(C,D)** Quantification of numbers of SA-β-gal^+^ cells **(A)** and numbers of p21^+^ cells **(B)**; values shown are mean ± SE from three experiments. **p < 0.01, one-way analysis of variance with Student’s *t*-test. **(E,F)** Western analysis of protein levels of p16 and p21 **(E)**, mTOR and p-mTOR **(F)** of cells treated with myelin and dimethyl sulfoxide (myelin), or myelin with clemastine (myelin + CLEM) or non-treated (control). GAPDH and γ-tubulin were served as loading controls for **(E,F)**, respectively. Ratio values were calculated as “p16 or p21/GAPDH,” “mTOR or p-mTOR/γ-tubulin,” and normalized to ratio of control. Values shown represent mean ± SE. *p < 0.05, **p < 0.01, *n* = 3. **(G)** Western analysis of protein levels of mTOR and p-mTOR in hippocampus of WT; APP/PS1 and APP/PS1 mice treated with clemastine; γ-tubulin was served as loading control.

A strong correlation between Aβ plaque and the senescence of OPCs surrounding it was observed in the APP/PS1 mice (Zhang et al., 2019). Selectively removing senescent OPCs reduces the Aβ deposition and ameliorates the cognitive deficits of the mice (Zhang et al., 2019), emphasizing a causal role of senescent OPCs in AD pathogenesis. Therefore, we wonder if the therapeutic effects of clemastine on modulating Aβ deposition and cognition are mediated by preventing the senescence of the glial cells. To directly measure the effects of clemastine on cellular senescence, we used an OLN cell line derived from primary glial cells (Richter-Landsberg and Heinrich, 1996). To induce cellular senescence, we added myelin debris as an external stressor to mimic the overwhelming degradation of myelin under the pathological condition that contributes to the senescence of glial cells (Safaiyan et al., 2016). Also, we measured the activity of the lysosomal enzyme β-galactosidase and the expression of p21, two markers of cellular senescence to detect the senescent cells (Lee et al., 2006; Jurk et al., 2012). The myelin debris induced OLN senescence, as high levels of β-galactosidase activity (SA-β-gal^+^) and the accumulation of p21 (p21^+^) were detected in a portion of the cells (Figures 6A–D). Interestingly, the treatment of clemastine significantly reduced both the number of SA-β-gal^+^ cells (Figures 6A,C) and p21^+^ cells (Figures 6B,D) to a basal level comparable with the non-treated control cells. Thus, clemastine can prevent the senescence of glial cells.

Previously, we reported clemastine enhances autophagy while suppressing the mTOR pathway *in vitro* and *in vivo* (Li et al., 2021), both of which exhibit strong interplay with cellular senescence (Kang et al., 2011; Kwon et al., 2017; Park et al., 2020). Especially, suppression of mTOR plays pivotal roles in preventing cellular senescence in a way either dependent or independent autophagy (Carroll and Korolchuk, 2017; Carroll et al., 2017; Park et al., 2020). Thus, we further examined whether clemastine altered mTOR activity in myelin debris-treated OPCs. Through Western blot analysis, we found myelin debris treatment not only induced the upregulation of p21 and p16, the markers of cellular senescence, but also increased the levels of phosphorylated mTOR (p-mTOR, Ser2448) (Figures 6E,F), indicating increased mTOR activity. In contrast, the treatment of clemastine reduced the levels of both p21 and p16, as well as p-mTOR to be comparable with the control (Figures 6E,F). Moreover, consistent with the previous observation (Li et al., 2021),we also found that both mTOR and p-mTOR levels were increased in the hippocampus of the APP/PS1 mice, whereas the treatment of clemastine reduced the protein levels to be comparable with the WT mice (Figure 6G). Therefore, clemastine prevents the senescence of glial cells and the upregulation of mTOR activity concomitantly.

**FIGURE 7:**
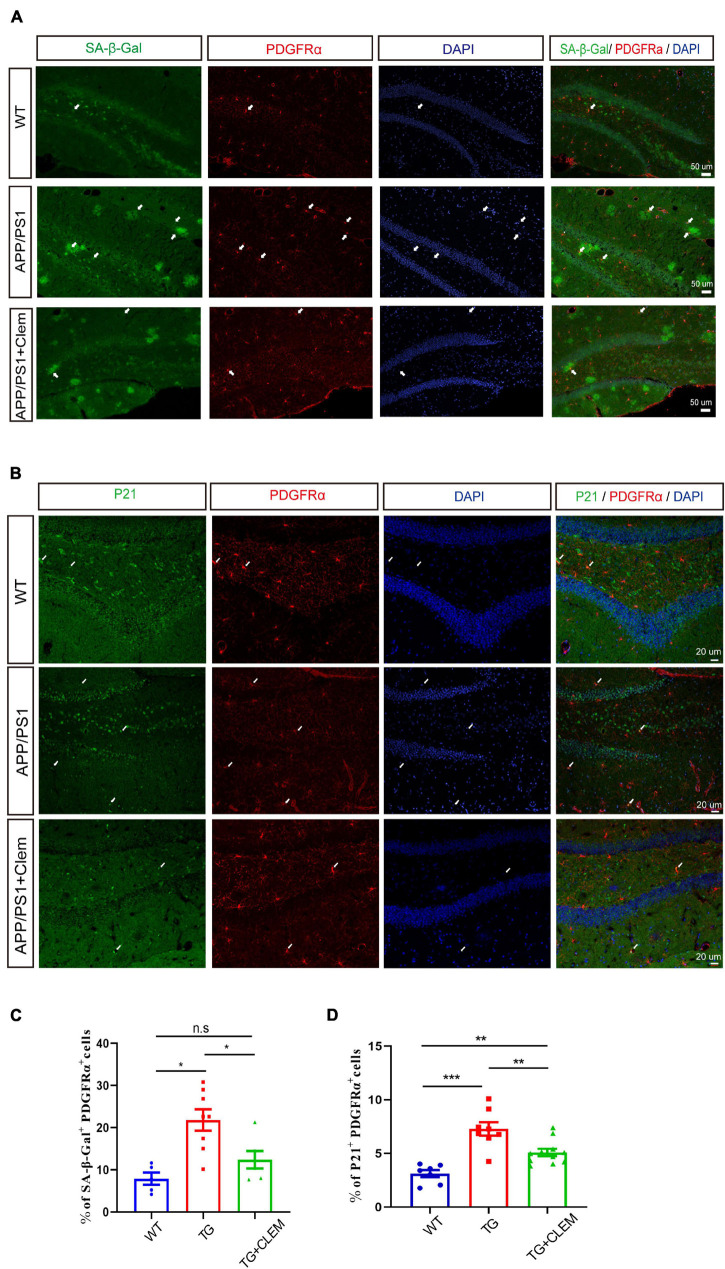
Clemastine treatment prevents senescence of OPCs in aged APP/PS1 mice brain. **(A,B)** Confocal images of immunolabeling hippocampal OPCs with PDGFRα (red) and 4,6-diamidino-2-phenylindole (blue) and SA-β-gal **(A)** or p21 (green) **(B)** in WT; APP/PS1 and APP/PS1 mice treated with clemastine. **(C,D)** Quantification of percentage of SA-β-gal^+^ PDGFRα^+^ **(C)** or p21^+^ PDGFRα^+^ **(D)** cells. Values shown are mean ± SE. *p < 0.05, **p < 0.01, ***p < 0.001, *n* = 3 mice per group, one-way analysis of variance with Student’s *t*-test.

To further corroborate the senescence prevention effect of clemastine on OPCs, we measured the number of PDGFRα^+^ OPCs that were positive for SA-β-gal or p21 in the hippocampus of the mice brain. In comparison with the WT mice, there was a significant increase in the percentage of PDGFRα^+^ OPCs that was positive for SA-β-gal (Figures 7A,C) or p21 (Figures 7B,D) for the APP/PS1 mice, whereas the treatment of clemastine significantly reduced the percentage of the cells (Figure 7). This result indicates that the OPCs in the APP/PS1 mice were more susceptible to be senescent, whereas clemastine can effectively protect the OPCs from senescence. Together, our data suggest that clemastine prevents the senescence of OPCs in the aged APP/PS1 mice, which may contribute to the prevention of Aβ deposition in the aged APP/PS1 mice brain.

## Discussion

The discovery of clemastine and other compounds showing muscarinic antagonist properties can induce OPCs differentiation, and myelin formation provided new approaches to tangle with the diseases featuring demyelination, including MS (Mei et al., 2014; Abiraman et al., 2015; Apolloni et al., 2016). The encouraging results from the clinical trial of clemastine for treating MS further substantiated the possibility to enhance remyelination during the neurodegeneration process (Green et al., 2017). Comparing to MS, the degeneration process of AD is rather gradual as prominent brain pathology, and the cognitive deficits commonly appear at the later stages of the disease. In this study, by using aged APP/PS1 mice instead of young mice that were used in the previous studies (Chen et al., 2021; Li et al., 2021), we believe it recaptures the features of AD at a later stage and enables more objective evaluation for the treatment (Huang et al., 2016; Denver et al., 2018). Our finding that chronic treatment of clemastine can enhance myelin formation and prevents myelin degeneration in the aged AD mice indicates that clemastine potentially could induce remyelination process even for the patients with abundant Aβ deposition at the advanced stages.

OPCs can generate mature myelinating OLs in both gray and white matter of the central nervous system throughout life (Huang et al., 2014; Guo et al., 2021). The dynamic changes of myelin are closely associated with cognition during both development stages and the various neurodegeneration conditions (Liu et al., 2016; Papuć and Rejdak, 2018; Bai et al., 2021). Therefore, our finding that the short-term memory is improved upon clemastine treatment is likely to be a direct result of the potentiated remyelination. However, we also found that memory retention and learning ability are not improved upon the treatment, which could be attributed to that the mice are aged. Because the cognitive deficits of APP/PS1 mice begin to manifest when they are 7–8 months old (Reiserer et al., 2007), it might be too late to intervene at this late stage. Supporting evidence comes from our previous study, as we showed that the chronic treatment of clemastine could improve the memory retention and learning ability of 7-month-old APP/PS1 mice (Li et al., 2021).

The enhanced remyelination could be a result of sustained OPCs proliferation and/or enhanced differentiation. In a previous study, it was the number of OLs, but not OPCs, that was increased upon the treatment of clemastine (Liu et al., 2016). In contrast, here we show that both OLs and OPCs were increased upon the treatment. The discrepancy could be attributed to the different models used in the studies, as they used mice that underwent 2 weeks of social isolation, which does not induce the loss of OPCs (Liu et al., 2016). Moreover, a recent study found that in toxin-induced focal demyelination, the proliferation of the migrated OPCs is a requirement for them to differentiate at the lesion site (Foerster et al., 2020). Blocking the cell cycle of OPCs *in vitro* resulted in a significant compromise of the differentiation of the cells (Foerster et al., 2020). Therefore, it is possible that clemastine supports the normal cellular activities of OPCs, including proliferation and differentiation, preventing the loss of the cells rather than enhancing the cellular activities under the neurodegeneration conditions.

Previous studies showed that the effects of clemastine on promoting OPCs differentiation is at least partially due to its antimuscarinic properties (Mei et al., 2016; Chen et al., 2021). However, the downstream effects of antagonizing the receptors that lead to OPC differentiation are still unknown. On the other hand, because OPCs are more susceptible to Aβ deposition associated with cellular senescence than other types of glial cells (Zhang et al., 2019), it is likely that the senescence of OPCs represents a major impediment for them to proliferate and differentiate under the pathological condition. Although our pieces of evidence show that clemastine has a strong effect on preventing the senescence of OPCs, it indicates that clemastine supports the normal activities of OPCs by preventing its senescence.

Previously, we found that clemastine can modulate autophagy in an environment-dependent manner, as it can enhance autophagy by inhibiting mTOR in the APP/PS1 mice but not in the WT mice (Li et al., 2021). The relationship between mTOR signaling, autophagy, and cellular senescence is yet to be examined under the neurodegeneration conditions (Kwon et al., 2017; Martínez-Cué and Rueda, 2020). Nevertheless, a large body of evidence suggests that hyperactive mTOR, autophagic impairments, and cellular senescence are involved in the pathogenesis of AD (Li et al., 2010; Jahrling and Laberge, 2015; Uddin et al., 2020; Carreno et al., 2021). In contrast, our pieces of evidence further strengthened the relationship between mTOR activity and the induction of cellular senescence under pathological conditions. Thus, it is possible that clemastine induces the upregulation of mTOR-mediated autophagy, which contributes to preventing the cellular senescence of OPCs.

Studies have shown that autophagy and cellular senescence are associated with the changes of Aβ deposition (Spilman et al., 2010; Tan et al., 2013; Zhang et al., 2019). Selectively removing the senescent OPCs alleviates the Aβ accumulation in the APP/PS1 mice (Zhang et al., 2019), and inhibiting mTOR signaling also reduces Aβ levels in another AD mice model (Spilman et al., 2010). Herein, we suggest clemastine has a combinational effect of both upregulating autophagic activities and preventing cellular senescence that results in the reduced Aβ plaques. This observation differs from a study that shows Aβ deposition was not altered upon the treatment of clemastine for the 8-month-old APP/PS1 mice (Chen et al., 2021). However, it is consistent with our previous study that shows Aβ deposition is reduced upon the chronic treatment of clemastine for 4 months of 4-month-old APP/PS1 mice (Li et al., 2021). To sum it all, our study provides a new perspective to examine the relationship between mTOR signaling, OPCs senescence, and remyelination under the neurodegeneration conditions featuring protein aggregations.

## Data Availability Statement

The original contributions presented in the study are included in the article/Supplementary Material, further inquiries can be directed to the corresponding author/s.

## Ethics Statement

The animal study was reviewed and approved by Institutional Animal Care and Use Committee of Soochow University.

## Author Contributions

Y-YX, D-eX, YT, RC, LL, and Q-HM designed the study. Y-YX, T-TP, and XH performed the experiments and analyzed the data. HC drafted the manuscript. WH and Q-HM revised the manuscript. All authors contributed to the article and approved the submitted version.

## Conflict of Interest

The authors declare that the research was conducted in the absence of any commercial or financial relationships that could be construed as a potential conflict of interest.

## Publisher’s Note

All claims expressed in this article are solely those of the authors and do not necessarily represent those of their affiliated organizations, or those of the publisher, the editors and the reviewers. Any product that may be evaluated in this article, or claim that may be made by its manufacturer, is not guaranteed or endorsed by the publisher.

## Footnotes

1
https://rsb.info.nih.gov/ij
