# Supplementary material for: Clemastine Ameliorates Myelin Deficits via Preventing Senescence of Oligodendrocytes Precursor Cells in Alzheimer’s Disease Model Mouse
Source: Front Cell Dev Biol. 2021 Oct 21;9:733945. doi: 10.3389/fcell.2021.733945 (PMC8567029; doi:10.3389/fcell.2021.733945)
Supplement: Supplementary file 1 [file Table_1.docx]

**Supplementary Table 1. The antibodies used in this study**

| **Antibody Specificity** | **Cat No.** | **Source** |
| --- | --- | --- |
| Aβ (6E10) | SIG-39320 | Convance |
| PDGFRα | AF1062 | R&D systems |
| APC (CC-1) | OP80-100 UG | Merck Millipore |
| Myelin Basic Protein | 808401 | Biolegend |
| Anti-Myelin Basic Protein Antibody (Degraded MBP) | 6B9255 | Merck Millipore |
| p21 | ab109199 | abcam |
| P16 | ab54210 | abcam |
| mTOR (7C10) | 2983S | Cell Signaling Technology |
| Phospho-mTOR (Ser2448) | 2971S | Cell Signaling Technology |
| GAPDH | FD0063 | Fude Biological Technology |
| γ-tubulin | T6557 | Sigma |
| Goat anti-Rabbit IgG (H+L) Highly Cross-Adsorbed Secondary Antibody, Alexa Fluor 555 | A-21429 | Invitrogen |
| Donkey anti-Goat IgG (H+L) Cross-Adsorbed Secondary Antibody, Alexa Fluor 488 | A-11055 | Invitrogen |
| Goat Anti-Mouse IgG (H+L) | 115-001-003 | Jackson ImmunoResearch Laboratories |
